# En Face and Cross-sectional Corneal Tomograms Using Sub-micron spatial resolution Optical Coherence Tomography

**DOI:** 10.1038/s41598-018-32814-3

**Published:** 2018-09-25

**Authors:** Yu-Tung Chen, Chia-Ying Tsai, Yu-Kuang Chiu, Ting-Wei Hsu, Lily Wei Chen, Wei-Li Chen, Sheng-Lung Huang

**Affiliations:** 1 Graduate Institute of Photonics and Optoelectronics, National Taiwan University, Taipei, Taiwan; 2 Department of Ophthalmology, National Taiwan University Hospital, Taipei, Taiwan; 3 Department of Ophthalmology, Fu-Jen Catholic University Hospital, New Taipei City, Taiwan; 4 School of Medicine, College of Medicine, Fu-Jen Catholic University, New Taipei City, Taiwan; 5 Department of Ophthalmology, College of Medicine, National Taiwan University, Taipei, Taiwan; 6 Department of Electrical Engineering, National Taiwan University, Taipei, Taiwan

## Abstract

Accurate diagnosis of corneal pathology and morphological identification of different corneal layers require clear delineation of corneal three-dimensional structures and en face or cross-sectional imaging of palisade of Vogt (POV), neovascularization (NV) or corneal nerves. Here we report a prototype of full-field optical coherence tomography (FF-OCT) system with isotropic sub-micron spatial resolution in the en face and cross-sectional views. It can also provide three-dimensional reconstructed images and a large field of view (FOV) by stitching tomograms side by side. We validated the imaging power of this prototype in *in vivo* rat and rabbit eyes, and quantified anatomical characteristics such as corneal layer thickness, endothelial cell density and the intensity profile of different layers. This FF-OCT delineated the ridge-like structure of POV, corneal nerve bundles, and conjunctival vessels in rat eyes. It also clearly identified the vessel walls and red blood cells in rabbit model of corneal NV. The findings provided by this FF-OCT are expected to facilitate corneal disease diagnosis and treatment.

## Introduction

The human cornea is an optically transparent structure that consists of five different layers: epithelium, Bowman’s layer, stroma, Descemet’s membrane, and endothelium. Corneal health and the corneal transparency that is critical for light transmission are supported by the limbus, a structure between the cornea and conjunctiva^1^. Corneal diseases are the second most common cause of visual loss^2,3^ and may involve different layers of the cornea, making the identification of the exact layer of pathology important in both diagnosis and management. Currently, *in vivo* confocal microscopy (IVCM) and anterior segment optical coherence tomography (AS-OCT) are the two major imaging modalities used to obtain en face and cross-sectional images of corneal lesions. The commercially available AS-OCT, Visante OCT of Zeiss, uses the time-domain optical coherence tomography (TD-OCT) configuration. The TD-OCT configuration with the point detector can not capture en face image and its axial resolution is not high enough to distinguish the layered cornea. Currently, AS-OCT based on the spectral-domain optical coherence tomography (SD-OCT) configuration with corneal lens usually lacks of lateral resolution (i.e. >2 μm), but can acquire the cross sectional images at relatively faster speed than that of the FF-OCT configuration. IVCM provides unique functions for non-invasive, *in vivo* assessment of ocular structures, such as the real time visualization of corneal layers at the cellular level^4–6^. Owing to its high lateral resolution of 0.6–2.0 µm, IVCM can be used to identify the cell density^7^ and the morphology of corneal diseases such as dry eye disease^8^, infectious keratitis^9^ and herpes zoster ophthalmicus^10^ in different corneal layers. However, IVCM is not enough to distinguish between corneal layers due to its low axial resolution of approximately 4 µm^11,12^.

Optical coherence tomography is a powerful imaging technique for examining corneal characteristics and quantifying the properties of corneal structures due to its micron-level axial resolution, which is determined by the specification of the light source^13^. Recent studies have quantified the layers of anterior cornea^14,15^ and visualized the morphology of posterior cornea^16,17^. Chen S. *et al*. have presented both en face and cross-sectional images with isotropic micron-level spatial resolution in *ex vivo* rat endothelium to determine its morphology and cell density by micro-optical coherence tomography^18^. To our knowledge, however, there has been no previous reference to OCT instruments that can provide the isotropic sub-micron spatial resolution necessary for detailed imaging of *in vivo* animal disease models. Furthermore, the reconstruction of three-dimensional images with optical consecutive frames based on the isotropic resolution is an important function that has not been widely explored.

In this study, we present not only en face and cross-sectional views in the XZ and YZ planes, but also volumetric images with isotropic sub-micron spatial resolution. We tested our imaging system in *in vivo* and *ex vivo* normal rat corneas, as well as *in vivo* normal rabbit corneas and diseased rabbit corneas with corneal NV. We also visually reconstructed the blood flow within *in vivo* rabbit stroma and the ridge-like morphology of POV.

## Results

### Morphological identification of normal rat cornea

**Figure 1:**
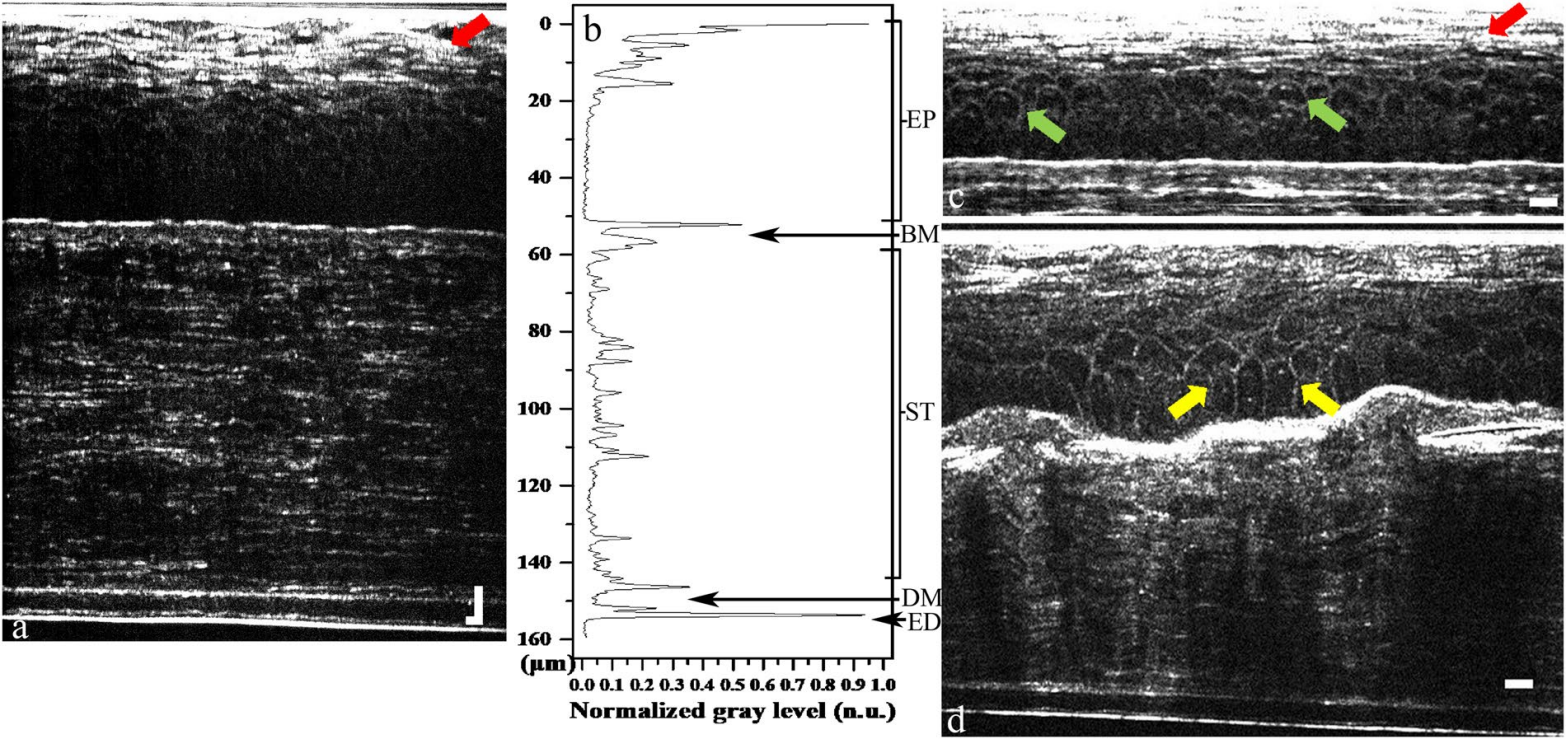
FF-OCT cross-sectional images of normal rat cornea. (**a**) Cross-sectional image of *in vivo* rat cornea. (**b**) Intensity profile along the axial direction in (**a**). (**c**) Rescaled cross-sectional image of *in vivo* rat corneal epithelial layer. (**d**) Rescaled cross-sectional image of *ex vivo* rat cornea. The red arrows mark non-keratinized squamous epithelial cells. The green arrows mark the cuboid wing epithelial cells. The yellow arrows mark columnar-shaped basal epithelial cells. EP: epithelium; BM: Bowman’s membrane; ST: stroma; DM: Descemet’s membrane; ED: endothelium. (Scale bar = 10 μm).

**Figure 2:**
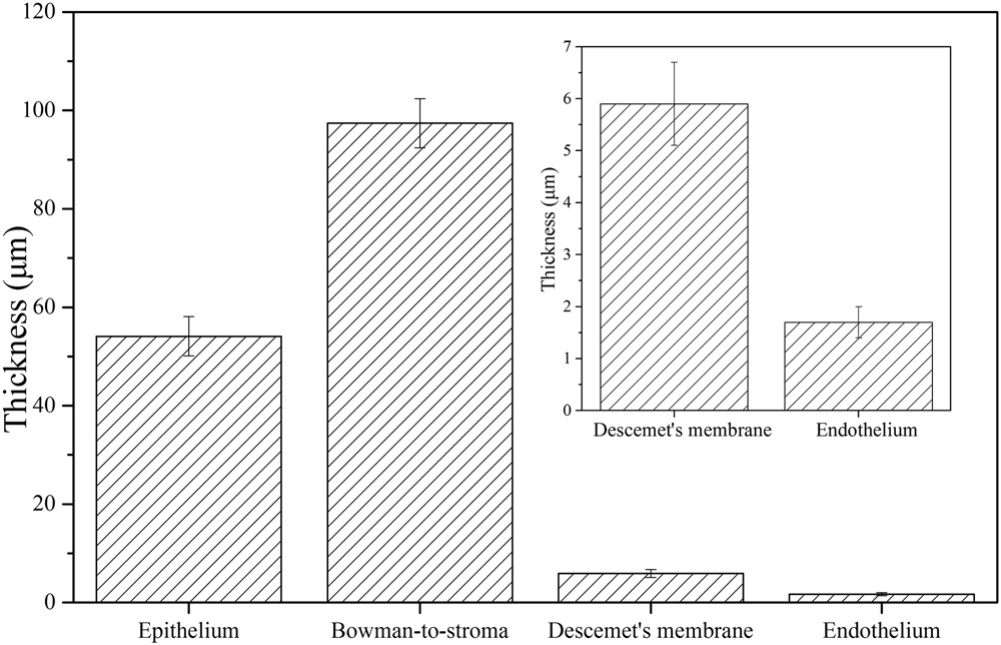
Average thicknesses of corneal layers.

*In vivo* cross-sectional FF-OCT imaging results of normal adult rat cornea showed clear interfaces between different corneal layers (Fig. 1a), as quantified by the corresponding corneal intensity profile. Each interface between adjacent layers represented one peak with ~0.9 μm of full-width at half-maximum (Fig. 1b). The average thicknesses of corneal epithelium, Bowman’s membrane-to-stroma, Descemet’s membrane, and corneal endothelium from Fig. 1 were quantified to be 54.1 ± 4.0 μm, 97.4 ± 5.0 μm, 5.9 ± 0.8 μm, and 1.7 ± 0.3 μm, respectively (Fig. 2). These thicknesses were defined as the peak-to-peak length in the intensity profile. Figures 1c,d demonstrate the morphological identification of three different layers within the corneal epithelium. Squamous superficial epithelial layer (red arrows in Fig. 1a,c) and cuboid wing epithelial cells (green arrows in Fig. 1c) could be identified easily in *in vivo* measurements. The *in vivo* identification of the cross-sectional cell boundary of the basal epithelial layer was more difficult, probably owing to the low index difference between cell membrane and the surrounding matrix as well as the reduced signal-to-noise ratio (S/N) from involuntary eye movement. We repeated the experiment with *ex vivo* enucleated eyes and could successfully delineate the columnar-shaped basal epithelial layer (yellow arrows in Fig. 1d).

**Figure 3:**
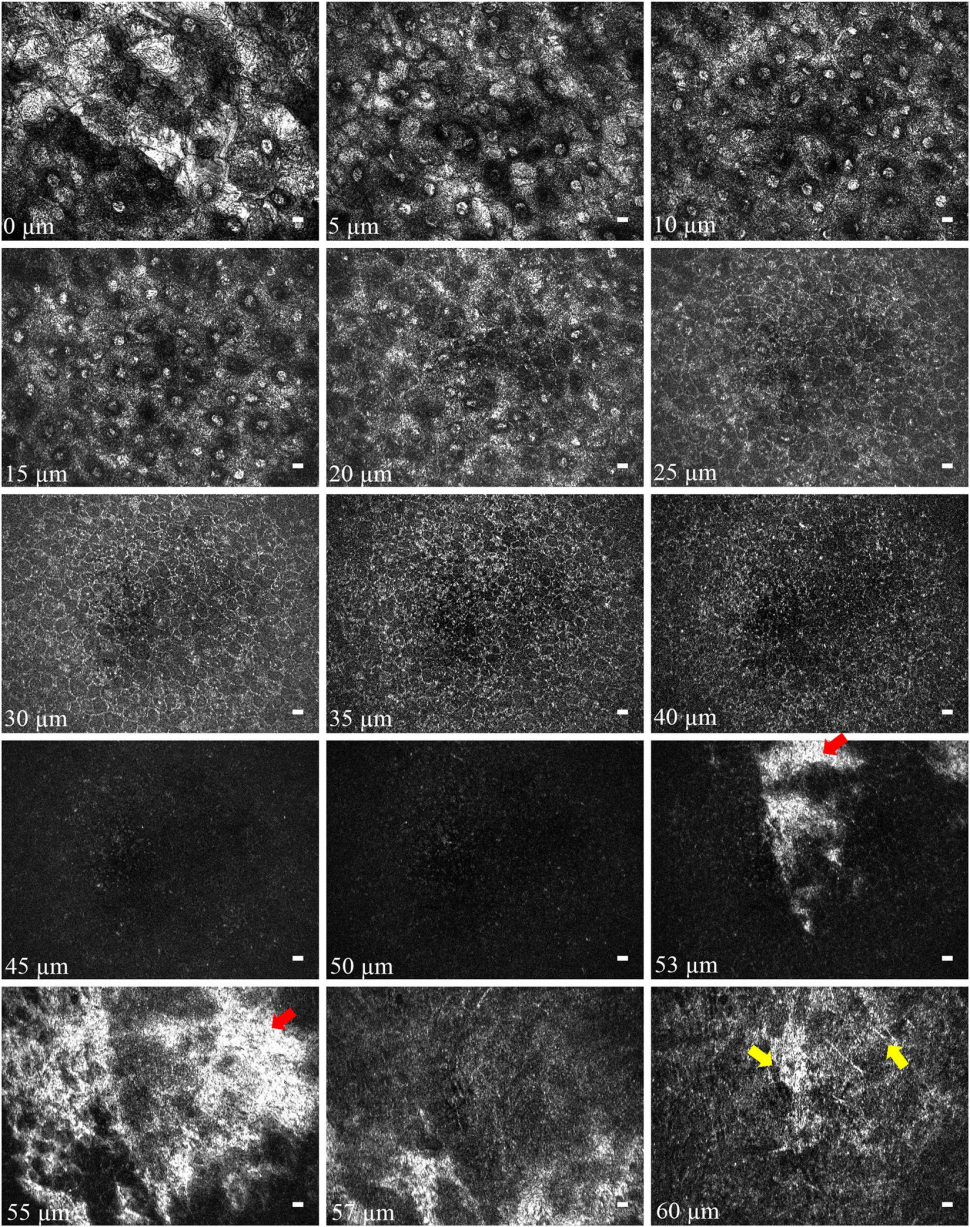
FF-OCT en face image sequence of *in vivo* normal rat cornea from the epithelial layer to anterior stroma. The following layers can be identified at the corresponding corneal depths: superficial squamous epithelial layer at 0–20 μm, superficial-to-wing transitional region at around 20 μm, wing layer at 20–40 μm, wing-to-basal transitional region at around 40 μm, and basal layer at 40–53 μm. The red arrows mark the interface between BM and epithelium. The yellow arrows mark the anterior stroma. (Scale bar = 10μm; value within each image corresponds to the depth in relation to corneal surface).

**Figure 4:**
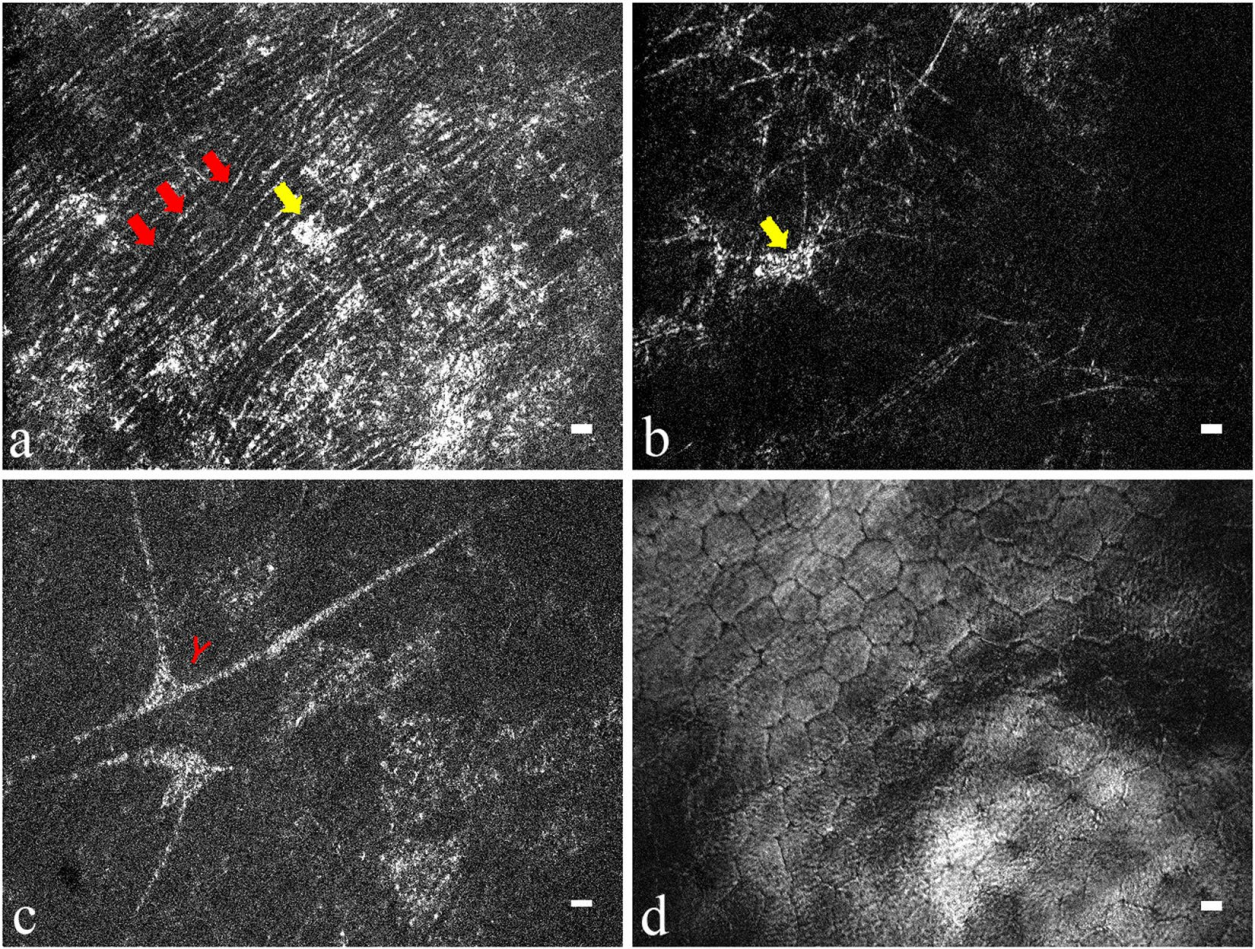
En face FF-OCT images of *in vivo* stromal and endothelial layers in normal rat cornea. (**a**) Anterior stroma. (**b**) Posterior stroma. (**c**) Nerve in stroma. (**d**) Endothelial layer. The red arrow marks collagen fibril in anterior stroma. The yellow arrows mark the stromal keratocytes. The red Y marks the corneal nerve bundle in the corneal stroma. (Scale bar = 10 μm).

With 0.2-µm interval of voxel depth, Fig. 3 shows the sequential results of en face imaging on *in vivo* rat corneas after a moving average filter of every five frames along the z (depth) direction. Images of superficial squamous epithelial cells, wing epithelial cells and basal epithelial cells were taken at depths of 0–20 μm, 20–40 μm, and 40–53 μm, respectively, from the corneal surface (Fig. 3). The interface between Bowman’s membrane (BM) and the epithelial layer was highly-reflective (red arrows in Fig. 3), possibly due to the different refractive indices. As seen from the yellow arrows in Fig. 3, the posterior side of BM (from 53- to 57-μm depth in Fig. 3) merges with the anterior stromal layer. Figure 4 presents the images of different layers of corneal stroma (Fig. 4a–c) and corneal endothelium (Fig. 4d). We identified the collagen fibril in anterior stroma (red arrows in Fig. 4a), keratocytes in anterior and posterior stroma (yellow arrows in Fig. 4a,b) and corneal nerve bundles in the corneal stroma (red Y in Fig. 4c). The cell density of polygonal endothelial cells was 2211 ± 166 per mm^2^.

**Figure 5:**
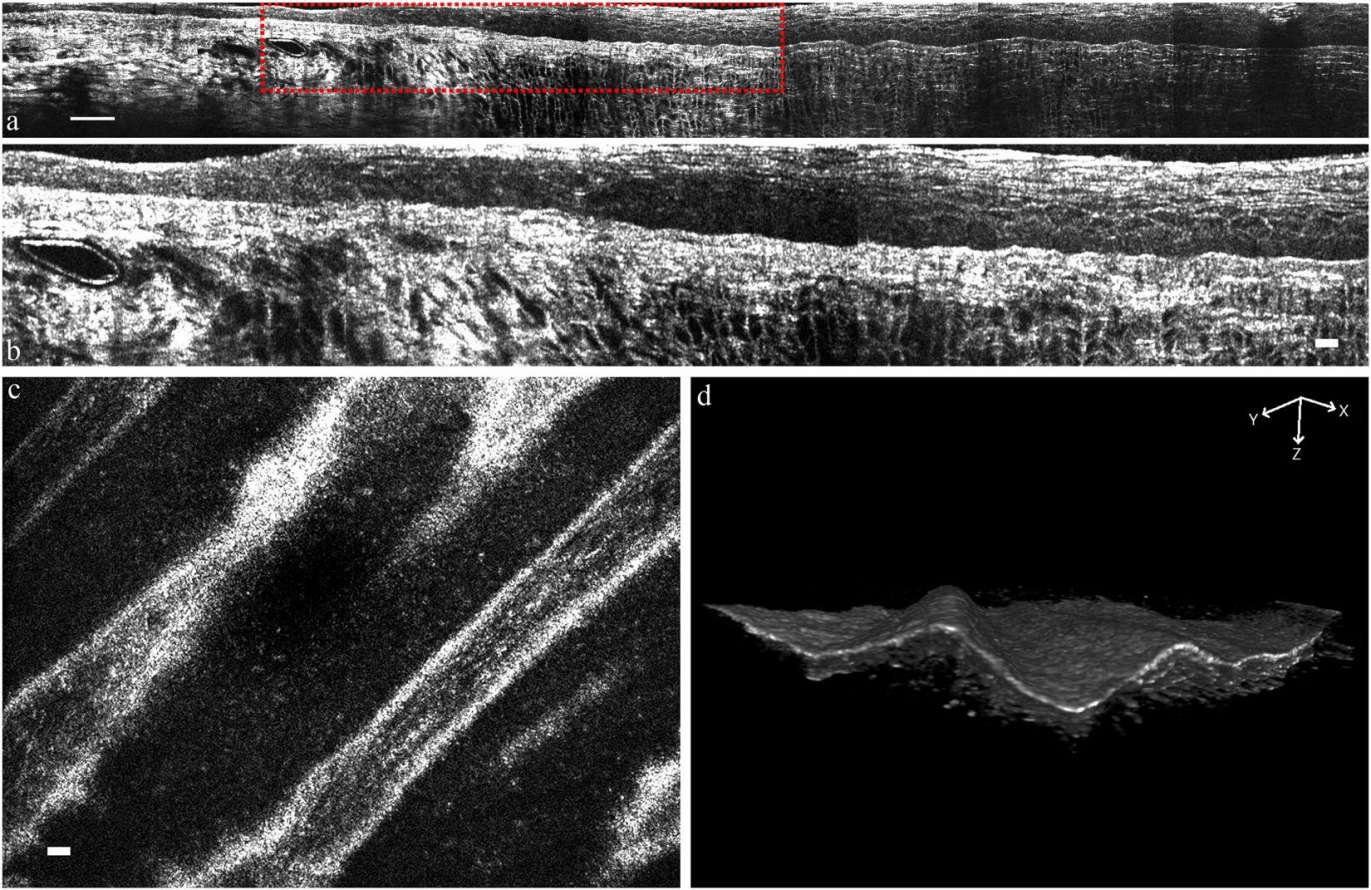
FF-OCT images of rat peripheral cornea to conjunctiva. (**a**) Rescaled *ex vivo* cross-sectional FF-OCT image reconstruction by composing seven consecutive tomograms from peripheral cornea to conjunctiva. (**b**) Magnified view of the region in (**a**) marked with red rectangle. (**c**) *In vivo* en face FF-OCT image of the normal rat’s POV. (**d**) *In vivo* reconstructed three-dimensional FF-OCT image of the normal rat’s POV. (Scale bar in (**a**) = 50 μm; scale bar in (**b,c**) = 10 μm).

Figures 5a,b show the consecutive *ex vivo* tomograms of cross-sectional images of the rat’s peripheral cornea, limbus, and paralimbal conjunctiva. The epithelial thickness and the number of layers in the cornea were significantly larger than those of the conjunctiva. Figure 5c demonstrates the *in vivo* en face images of rat’s POV and shows the clear finger-like projection of POV that is located within the limbal area between the conjunctiva (left side of Fig. 5b) and the cornea (right side of Fig. 5b). With the stacking of en face frames, we reconstructed the three-dimensional ridge-like POV tomogram (Fig. 5d). The consecutive tomograms of en face images of the rat’s peripheral cornea can be seen in the supplemental video 1, which is playable at a speed of 4-μm depth per second.

### Morphological identification of normal rabbit cornea

**Figure 6:**
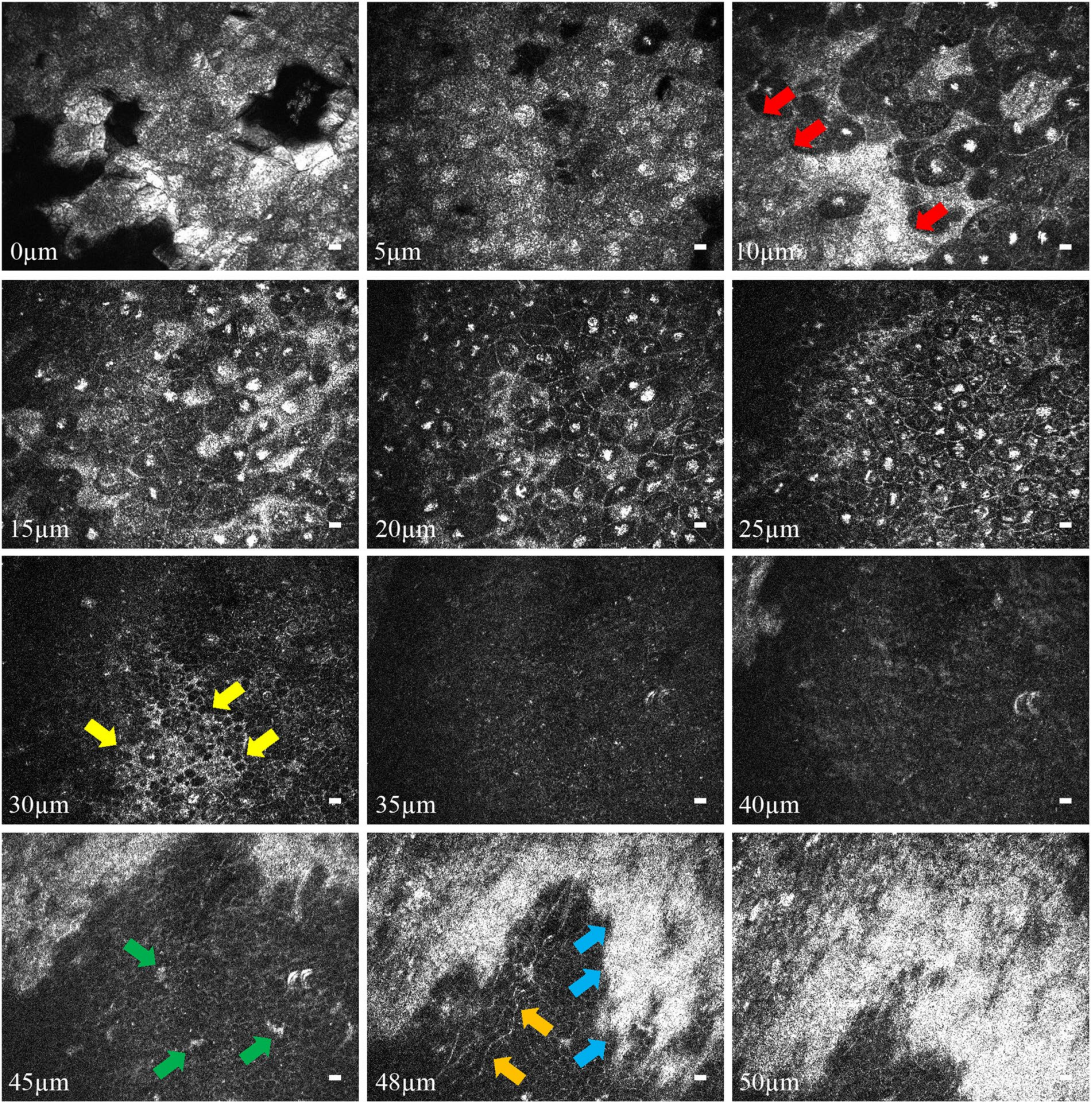
FF-OCT en face image sequence from the epithelial layer to Bowman’s membrane of *in vivo* rabbit cornea. The following layers can be identified at the corresponding corneal depths: superficial epithelial layer at 0–10 μm, superficial-to-wing transitional region at around 10 μm, wing layer at 10–30 μm, wing-to-basal transitional region at around 30 μm, and basal layer at 30–48 μm. The red arrows mark the interface between superficial and wing layers. The yellow arrows mark the interface between wing and basal layers. The blue arrows mark the interface between basal layer and Bowman’s membrane. The green arrows mark corneal inflammation with suspected dendritic cells. The orange arrows mark the nerve plexus. (Scale bar = 10 μm; value within each image corresponds to the depth in relation to corneal surface).

The en face images of *in vivo* rabbit cornea clearly show the three different epithelial layers (superficial squamous, wing cell, and basal epithelial layers) and the basement membrane (Fig. 6), which are similar to the results from normal rat cornea (Fig. 3). Sub-basal nerve plexus (orange arrows) and suspected dendritic cells (green arrow) were also identified.

### Morphological identification of vessels and red blood cells

**Figure 7:**
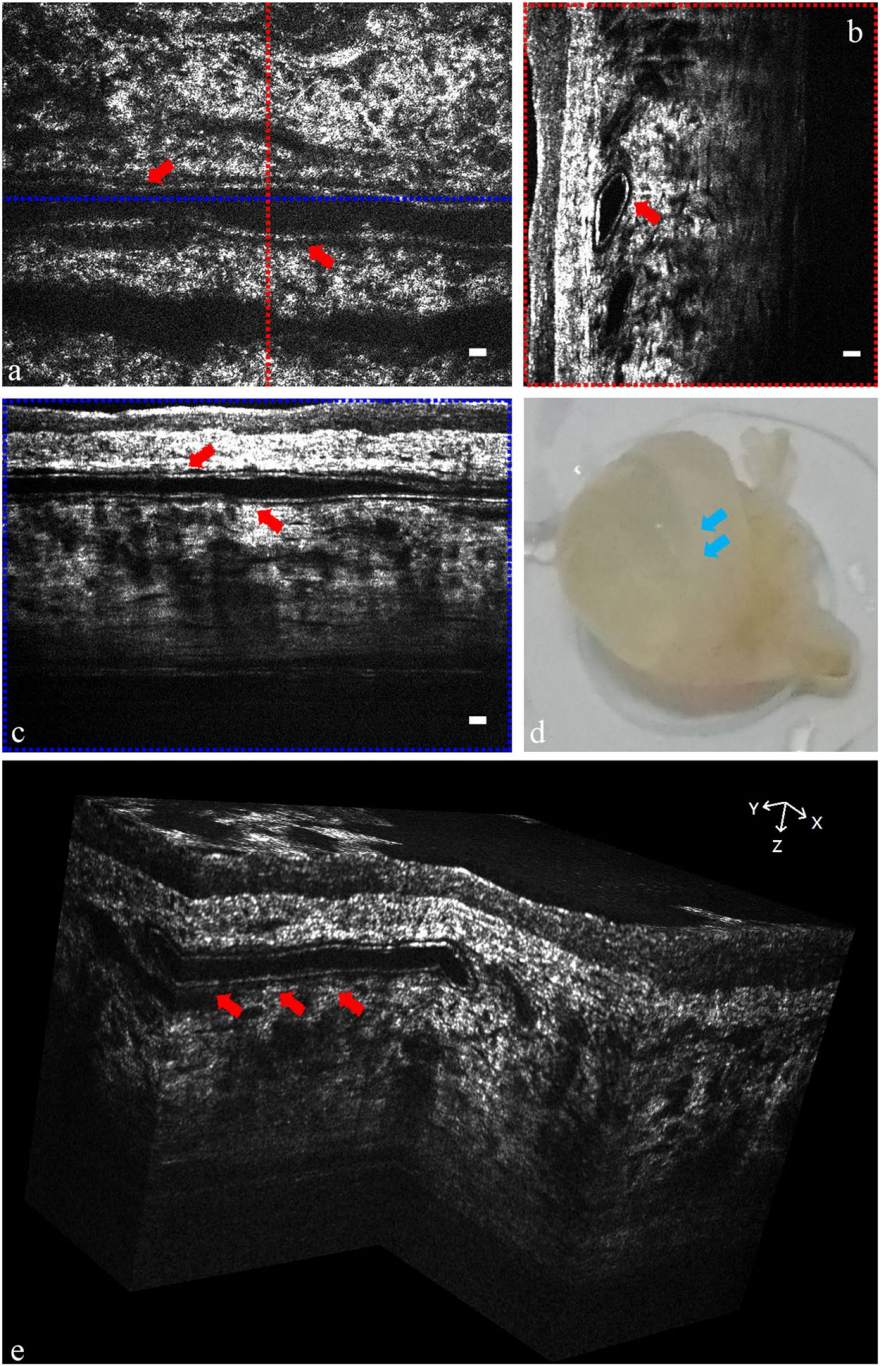
Images of normal *ex vivo* rat conjunctival vessels. (**a**) *E*n face image (XY) of normal rat conjunctiva vessel. (**b**) Rescaled cross-sectional image (YZ) corresponding to the red position in (**a**). (**c**) Rescaled cross-sectional image (XZ) corresponding to the blue position in (**a**). (**d**) One *ex vivo* rat eye specimen. (**e**) *Ex vivo* reconstructed three-dimensional FF-OCT image of rat conjunctiva vessel. The red arrows mark the vessel wall. The blue arrows mark the rat conjunctival area. (Scale bar = 10 μm).

**Figure 8:**
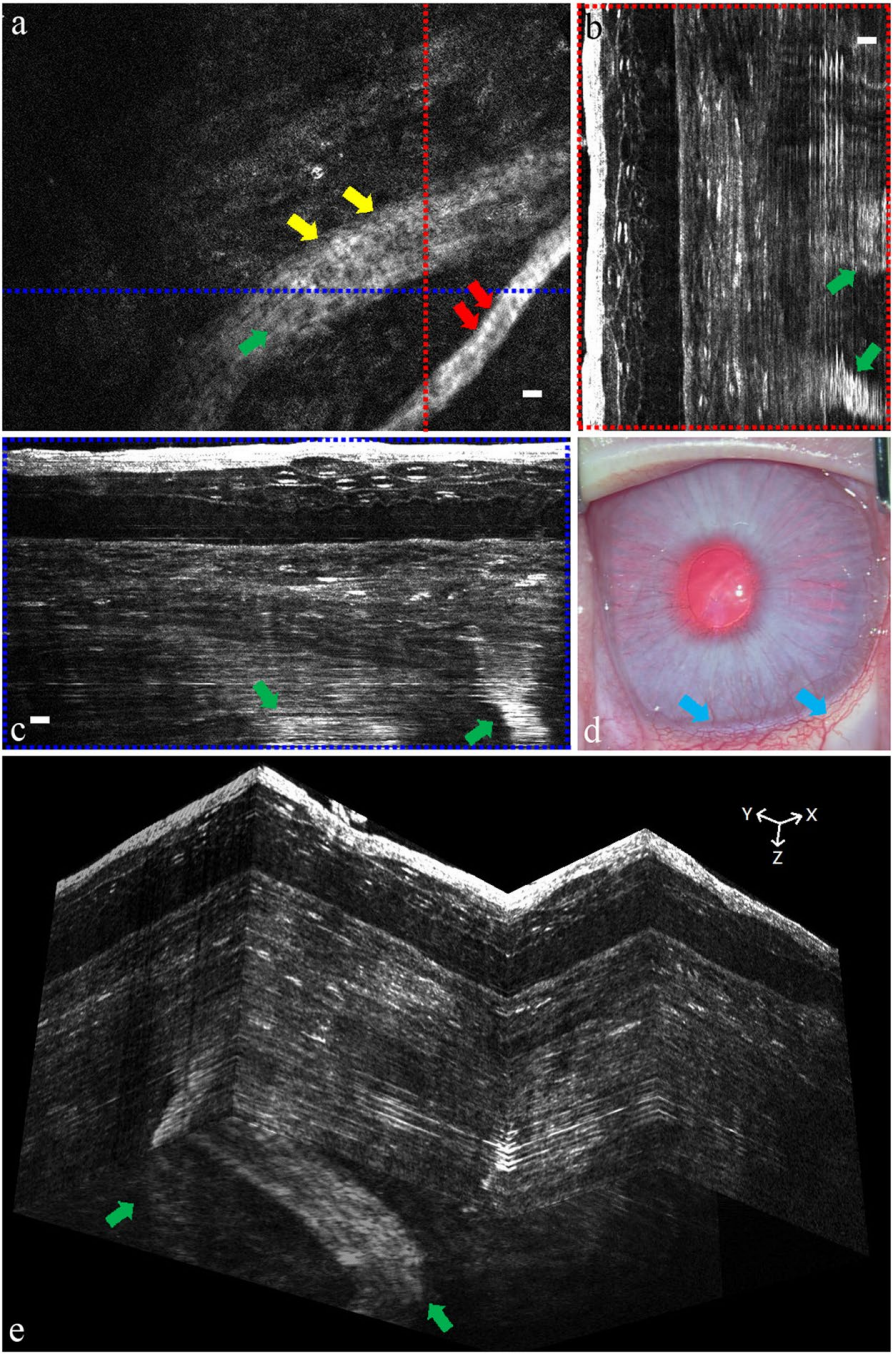
*In vivo* FF-OCT image of rabbit NV cornea. (**a**) *E*n face image (XY) of rabbit NV cornea. (**b**) Rescaled cross-sectional image (YZ) corresponding to the red position in (**a**). (**c**) Rescaled cross-sectional image (XZ) corresponding to the blue position in (**a**). (**d**) The gross view of rabbit NV cornea. (**e**) *In vivo* reconstructed three-dimensional FF-OCT image of rabbit NV cornea. The yellow arrows mark the vessel wall. The red arrows mark the red blood cells. The green arrows mark the vessel flow. The blue arrows mark the vessels. (Scale bar = 10 μm).

Dynamic behavior of the cornea could be observed because of the high resolution and high frame rate of the FF-OCT system. *Ex vivo* scans over normal rat conjunctival vessels and *in vivo* examinations of rabbit eyes with induced corneal NV were performed. The vessel walls from the normal rat conjunctiva and the rabbit NV cornea (red arrows in Fig. 7 and yellow arrows in Fig. 8) were identified in the en face, cross-sectional, and three-dimensional views. As shown in Fig. 8, red blood cells (red arrows) and blood flow (green arrows) were clearly identified in the *in vivo* examination of rabbit eyes with induced NV formation. The blood flow in the corneal NV was hyporeflective in FF-OCT cross-sectional images owing to the flowing effect and vessel movements. Supplemental video 2 shows the flow of red blood cells within the blood vessel in the stromal layer.

## Discussion

The nearly isotropic high resolution and fast imaging rate of both en face and cross-sectional views enabled the acquisition of the fine anatomical details of the cornea and its dynamic behavior. FF-OCT has been used to diagnose corneal diseases in human donor and pathological corneas via the capturing of en face images^19^.With the crystalline fiber based light source and the Mirau technique in our FF-OCT system, however, detailed cross-sectional information and *in vivo* scanning results were further delineated. The crystalline fiber offers very broad emission for high depth resolution; while the Mirau objective NA provides high lateral resolution. For corneal lesions that can be seen in the en face view, our system has the potential to show their specific depths in the cornea with the cross-sectional view. With this advantage, different corneal dystrophies can be observed in detail for better diagnosis accuracy. In patients with recurrent corneal erosion, high image quality is important for ophthalmologists to locate the diseased basement membrane and apply YAG laser with accuracy. In some atypical but vision-threatening corneal infections such as Acanthamoeba, fungal, or microsporidial keratitis, the high 3D-resolution FF-OCT may provide early diagnosis and recurrence detection. Quantitative information such as cell density or cytoplasmic and nucleus ratio can be extracted readily with the high-resolution images of endothelial cell and inflammatory cells. Our system can also provide other quantitative data, including epithelial cell density, cell size, nerve density in different layers. Due to the high en face rate, our FF-OCT can also provide dynamic parameters, such as blood flow speed. In this study, we captured clear images of POV, corneal nerves, corneal neo-vessels, and normal conjunctival vessels which are helpful in the diagnosis and treatment of limbal insufficiency, neurotrophic ulcers, ghost vessels or corneal NV.

The FF-OCT is a kind of TD-OCT but with a much fast imaging speed due to the 2D CCD camera, which serves as the detector of the interferometric signal. In our FF-OCT system, the PZT scanning speed can achieve 13 μm/sec (i.e. limited by the camera frame rate); while the OCT en face frame rate can reach 65 frame/sec. The 3D imaging reconstruction was done in real time. Despite the fast imaging speed, it does come with a drawback of reduced dynamic range due to the quite limited full well electron capacity of the CCD pixel as compared to a typical point detector. IVCM is now widely used in cornea clinics to detect the layered corneal structure and measure the endothelium cell density. IVCMs have good lateral resolution but the depth resolutions are not enough to precisely distinguish the layers of cornea. For both IVCM and FF-OCT, the signals come from the back-scattered light. So, in principle, the acquired images should be highly correlated except the spatial resolutions. With the isotropic spatial resolution, more cornea features can be resolved, such as the 3D nerve network. SD-OCT has the merit of fast imaging speed for acquiring cross-sectional images, while the FF-OCT could generate the en face images in real time and with better lateral resolution. To obtain a 3D tomogram, the imaging speed of FF-OCT is in general faster than that of SD-OCT due to the fast advancing CCD or CMOS cameras in terms of pixel numbers and frame rate. With some modification, our OCT system can be improved to image greater penetration depth to around 700 μm for detecting human corneas with edematous change. Replacing the present PZT (~400-µm traveling distance) with greater than 700-µm traveling distance, which is now commercially available, is one possibility. At such a deep penetration, the light dispersion in cornea may need a more sophisticated compensation to avoid deteriorated depth resolution. This could be done by fine-tuning the index of refraction of the silicon oil in our Mirau objective.

Reducing S/N ratio is needed for obtaining satisfactory OCT images. In this study, the animals were anesthetized during scanning, and in the real world for future clinical use, patients may not keep still better than these sedated animals. Higher camera frame rate can mitigate the S/N ratio issue due to involuntary eye movement. However, higher light source power is needed for higher camera frame rate, which could potentially cause bio-toxicity. Therefore, we stay with the present imaging speed to avoid damaging the sedated animals. Another way to improve the S/N ratio is to increase the interference efficiency (IE) of the Mirau interferometer. The present IE is near 50%. So there is a 3-dB range to improve the S/N ratio. To increase the IE, the stray light (i.e. non-interference light) from the Mirau objective needs to be reduced. This may be done using a highly selective spatial frequency filter at the expense of a significantly enlarged FF-OCT system.

In conclusion, this study uses the crystalline fiber based light source and a specially tailored Mirau objective. The former enables the realization of submicron depth resolution with low image pixel cross talk because of the broad and near Gaussian spectrum; while the latter facilitates the compact interferometer design and also eliminates the need for identical high numerical aperture objective pair in a conventional Michelson interferometer configuration. The FF-OCT system we designed has a unique and fast scanning method for the concurrent display of en face and cross-sectional views with three-dimensional tomogram. Assessment of *in vivo* animal models and *ex vivo* animal specimens gave results that were consistent with medical literature. This study successfully captured the *in vivo* morphology of various cells and identified the rabbit NV cornea and the large FOV that extended from the conjunctiva to the cornea. In the future, we plan to broaden the application of our system and use it to assess human endothelium. The assessment of human endothelium, which is located at a corneal depth of approximately 700 µm and deeper than our current imaging depth, can be especially helpful for cases of edematous cornea.

## Methods and Materials

### Experimental setup, system performance and image acquisition

The experimental setup of our FF-OCT with sub-micron spatial resolution was modified from our previously reported system for cornea measurement with long penetration depth^13^. The broadband light source of the FF-OCT was made from Ce^3+^:YAG single-cladded crystal fiber, pumped by a 1-W, 445-nm laser diode. The center wavelength and bandwidth are respectively 560 and 95 nm, giving the OCT system a high axial resolution of 0.9 µm. To characterize the system depth resolution, the interface between a glass plate and air was measured to analyze it. A typical interference pattern is shown in supplemental data 1. To enhance the silica fiber coupling, the crystal fiber’s cylindrical shape was designed to effectively remove heat to increase the brightness of the broadband emission. Light was delivered to the FF-OCT system by a 400-µm-core multimode fiber. Since the S/N ratio of the FF-OCT system is proportional to the average power of the light source, the bright continuous-wave emission of the crystal fiber source is more advantageous for clinical applications as compared with pulsed light sources. To minimize external disturbances such as environmental vibration and sample movement, a Mirau-based OCT configuration was adopted. The Mirau objective was used to illuminate the cornea subject and collect the back-scattered light from the subject and the reference arm. To compensate for the chromatic dispersion of the subject, the Mirau objective was water immersed. For large FOV and near-isotropic spatial resolution, a 20 × water-immersion objective lens (Olympus, UMPlanFLN 20 × W, NA: 0.50) was employed to achieve a lateral resolution of 0.8 μm and the distance of 10%-90% signal from a glass interface was measured as shown in supplemental data 2. Our PZT scanning speed can achieve 13 μm/sec (i.e. limited by the camera frame rate); while the OCT en face frame rate can reach 65 frame/sec. The maximum achievable FOV of the 20X objective is 1 mm × 1 mm. In addition, the XY scanning stage (Thorlabs, MLS203-1) was installed to compose a large area by stitching several tomograms, each with a FOV of 292 μm × 220 μm (x × y). To view the *in vivo* rabbit corneal NV, a 400-μm-long piezo-electric transducer (PZT) (PI, #P-725.40 L) with open-loop control was used. Each tomogram acquired by our FF-OCT system was stored by stacking en face frames with 0.2-μm separation. For 3D reconstruction, the 3D images were built in real time by stacking the en face images using the ImageJ software. In general, 3D moving-average imaging processing was performed before reconstructing the 3D images. The 3D moving-average processing was done by setting a pixel with a radius first, and then the pixels within the spherical volume associated with each selected pixel were averaged. In this study, we provided two scale bars for the cross-sectional image in Fig. 1a. To avoid the distortion of cell morphology, we rescaled the axial scale to create cross-sectional isotropic images in Figs 1c, 4, 6 and 7.

### Animal model and sample preparation

We assessed normal healthy rat and rabbit corneas and a disease model of rabbit corneal NV in this study. Adult male rats (Sprague Dawley, 0.25–0.3 kg, 3 months old) and adult male New Zealand white rabbits (3.0–3.5 kg, 6 months old) were used. The use, care, and treatment of all animals were in strict agreement with the ARVO Statement for the Use of Animals in Ophthalmic and Vision Research. All experimental procedures were approved by the Committee for Animal Research of the National Taiwan University Hospital. All *in vivo* procedures for animals were performed under general anesthesia induced by intramuscular injection of zoletil (0.25 ml/kg) and rompun (0.2 ml/kg), and the examined eyes were topically anesthetized with 0.5% proparacaine hydrochloride (Alcain, Alcon, TX) before manipulation. The right eye of each animal was used for the experiments. For *ex vivo* examination, animals were sacrificed with an overdose of potassium chloride (5 ml/kg) injection into the marginal ear vein or heart. During FF-OCT scanning, the vertical area was from the top of epithelial layer to the bottom of endothelial layer; the horizontal area was from the nasal conjunctiva to the temporal conjunctiva, each side has a 2-mm margin from the limbus.

To create a stable and extensive corneal NV model in rabbit eyes, we modified the closed eye contact lenses (CLs) method described by Chen, *et al*.^20^. Corneal NV was created by placing hard CLs made of poly(methyl methacrylate) (PMMA, Boston; 7.6-mm base curve, 13-mm diameter) onto the right eyes of New Zealand white rabbits. To maintain the lenses in place, 5–10 interrupted 5–0 silk sutures (Ethicon; Somerville, NJ) were sutured through the superficial tarsus of both eyelids with care to avoid penetrating or damaging the tarsal conjunctiva. We closed about 80–90% of eyelid fissures with tarsorrhaphy. An operative microscope (OPMI Pico I; Carl Zeiss Meditec, Jena, Germany) was used to record the growth of corneal NV. In this model, corneal NV occurred within 1 week after the induction, peaked at 5 to 7 weeks after CLs wear and regressed gradually or remained stationary thereafter. We examined the eyes at 6 weeks after the induction of corneal NV.

### Statistical analysis

All data analyses were computed by the MATLAB and ImageJ software. For the *in vivo* corneal analysis, average thickness of the layers within cornea were quantified based on more than 100 random positions. Of these, ten were selected from each three-dimensional OCT tomogram and the mean ± standard deviation (SD) was presented. Additionally, cell density of corneal endothelium was evaluated by averaging the surface area of images with the corresponding amount of corneal endothelial cells.

## Electronic supplementary material

Supplemental data

Supplemental video 1

Supplemental video 2
