# Supplementary material for: En Face and Cross-sectional Corneal Tomograms Using Sub-micron spatial resolution Optical Coherence Tomography
Source: Sci Rep. 2018 Sep 25;8:14349. doi: 10.1038/s41598-018-32814-3 (PMC6156507; doi:10.1038/s41598-018-32814-3)
Supplement: Supplementary file 1 — Supplemental data [file 41598_2018_32814_MOESM1_ESM.pdf]

En Face and Cross-sectional Corneal Tomograms Using Sub-micron spatial resolution Optical Coherence Tomography

Yu-Tung Chen[1], Chia-Ying Tsai[2,3,4], Yu-Kuang Chiu[1], Ting-Wei Hsu[1], Lily Wei Chen[2], Wei-Li Chen[2,5]* & Sheng-Lung Huang[1,6]*
*Co-corresponding authors

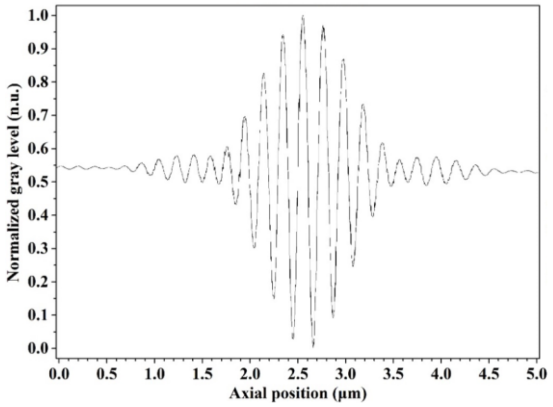

(a)

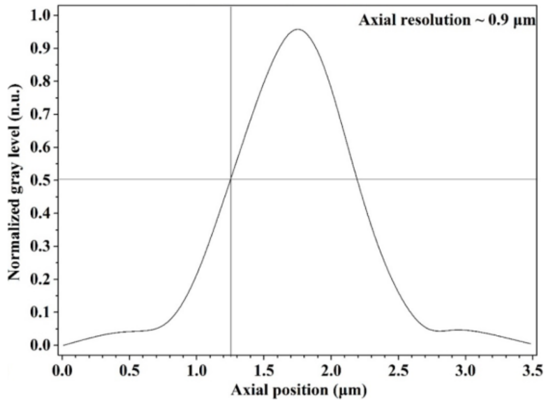

(b)

Supplemental data 1: (a) A typical interference pattern and (b) its envelop from the interface between a glass plate and air

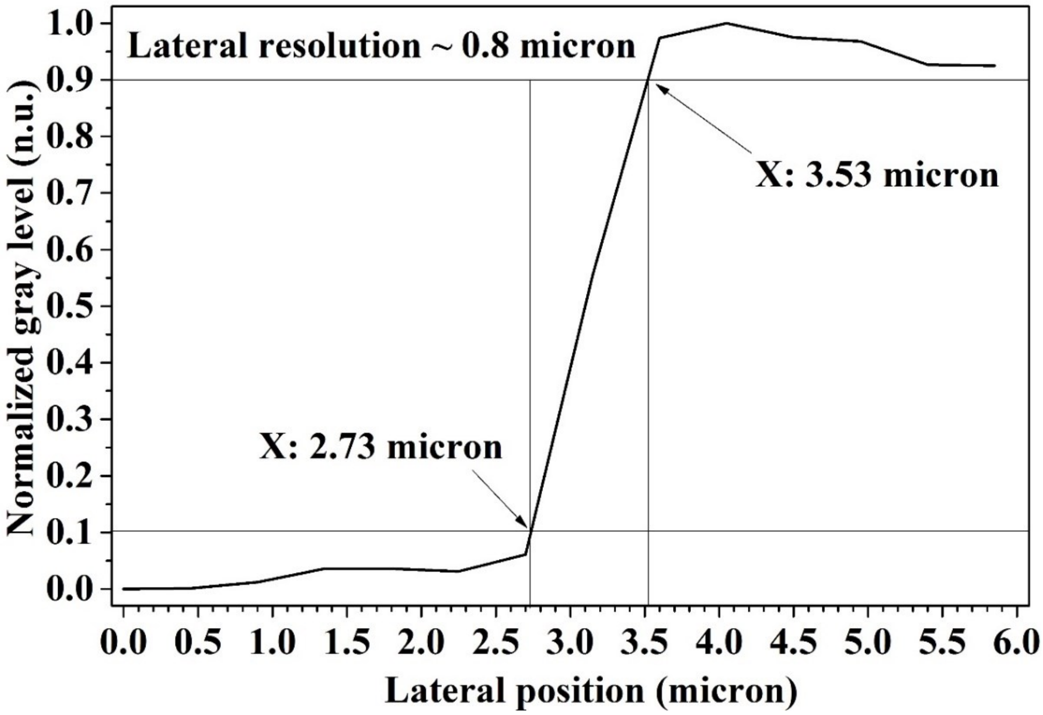

Supplemental data 2: The lateral resolution measurement using a glass plate edge
